# Impact of carbon content on the adsorptive performance of Zr-MOF composites for diclofenac sodium removal

**DOI:** 10.1039/d5ra04089b

**Published:** 2025-10-27

**Authors:** Sherif Hegazy, Konsta Saaranen, Tao Hu, Sari Tuomikoski, Ulla Lassi, Varsha Srivastava

**Affiliations:** a Research Unit of Sustainable Chemistry, University of Oulu P. O. Box 4300 FI-90014 Oulu Finland varsha.srivastava@oulu.fi +358414712348; b Kokkola University Consortium Chydenius, University of Jyväskylä FI-67100 Kokkola Finland

## Abstract

Efficient removal of pharmaceutical pollutants such as diclofenac sodium (DCF) from water is essential for reducing environmental contamination. This study explores the effect of biomass-derived carbon content on UiO-66-NH_2_-based Zr-MOF composites for DCF removal, using a simple one-pot synthesis. The work introduces a sustainable and low-cost strategy by valorizing industrial waste wood into functional carbon. Structural and surface characterization (XRD, SEM, TEM, FTIR, and XPS) confirmed successful integration of carbon into the MOF framework. Adsorption experiments revealed that composites with 10–20% carbon content offered the best performance, with Zr-MOF@C-10% achieving the highest adsorption capacity (*q*_m_ = 385 mg g^−1^), particularly at DCF concentrations exceeding 300 mg L^−1^. Although moderate carbon addition enhanced microporosity and maintained relatively high surface area, higher carbon loading (40–60 wt%) led to reduced surface area and possible pore blockage, limiting adsorption efficiency. The adsorption mechanism involves electrostatic interactions, hydrogen bonding, and π–π stacking, reflecting the synergistic contribution of surface functional groups and pore structure. These findings demonstrate the potential of biomass-derived Zr-MOF@C composites as effective and sustainable adsorbents for pharmaceutical removal from water.

## Abstract 2

Efficient removal of pharmaceutical pollutants such as diclofenac sodium (DCF) from water is essential for reducing environmental contamination.

## Graphical abstract

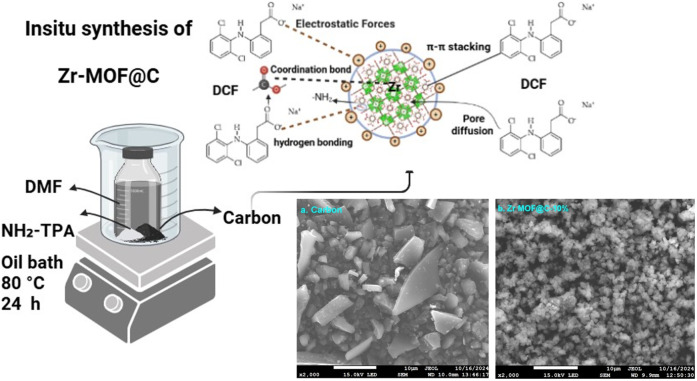

## 1 Introduction

The presence of pharmaceuticals in the water environment was first identified in the 1970s and remains a significant concern due to their persistence in ecosystems and potential risks to potable water quality.^1^ Among the wide range of pharmaceuticals, non-steroidal anti-inflammatory drugs (NSAIDs), analgesics, and antibiotics are frequently detected in aquatic environments, particularly in domestic effluents.^2,3^ Among these, diclofenac sodium (DCF), a widely used NSAID, stands out due to its extensive global usage, estimated at 940 tons annually.^4^ Around 65% of the orally administered dose is excreted in urine along with active metabolites, eventually reaching conventional wastewater treatment plants. DCF is frequently detected in surface and wastewater, often exceeding the European Commission's environmental quality standards (EQS) of 0.1 μg L^−1^, indicating significant ecotoxicological risks.^5,6^ Efforts to mitigate the environmental impact of DCF have highlighted the need for effective removal methods. Various approaches, including advanced oxidation,^7^ biodegradation,^8^ irradiation,^9^ and adsorption have been explored for the treatment of pharmaceuticals from water.^10^ Among these, adsorption stands out as a cost-effective and scalable solution, appreciated for its simplicity and feasibility in large-scale applications.^11^

Adsorbents such as activated carbon, biochar, and metal–organic frameworks (MOFs) have shown significant potential for the removal of pharmaceutical pollutants, including DCF. However, while activated carbon offers high surface area and adsorption capacity, its regeneration and reuse remain challenging. Recent research has focused on the use of MOFs, such as Zr-based MOFs, for their tunable porosity, high surface area, and functionalized surfaces, which enhance their adsorption properties for pharmaceutical contaminants.^12,13^ For instance, He *et al.*^14^ found that Bi-MOFs exhibited excellent adsorption and photocatalytic properties for DCF removal. Similarly, Hasan *et al.*^15^ demonstrated that functionalizing UiO-66 with SO_3_H groups resulted in a remarkable enhancement of adsorption kinetics and capacity for DCF. Furthermore, dos Reis *et al.*^16^ reported that selenium-doped biochar achieved effective DCF removal, with adsorption capacities reaching 355 mg g^−1^ through multiple mechanisms such as pore filling, π–π interactions, and hydrogen bonding between the biochar-Se composite and DCF.

The integration of carbon materials with UiO-66 frameworks enhances their properties, such as surface area, porosity, and functional group availability, which improve adsorption through hydrogen bonding and electrostatic interactions. For instance, activated carbon-UiO-66 composites exhibit superior adsorption capacities for volatile organic compounds due to their enhanced surface characteristics.^17^ Additionally, functional groups like Zr–O–C bonds, as in the gGO-U-50 composite, significantly improve pollutant-specific interactions, demonstrating effective fluoride removal.^18^ Building on this, a recent work optimized the surface charge of UiO-66-NH_2_@HTC composites through acidic and basic treatments, achieving improved dye adsorption and highlighting the benefits of tailored surface modifications.^19^ Despite these advancements, the precise carbon content required for optimal performance in MOF composites remains an area of active investigation.

This study explores the effect of biomass-derived carbon content on UiO-66-NH_2_-based Zr-MOF composites for DCF removal, using a simple one-pot synthesis without specialized equipment. The work introduces a sustainable and low-cost strategy by vaporizing industrial waste wood.

## 2 Experimental section

### 2.1. Materials

The carbon sample used in this work was provided by Karelian Paju Company, Finland. This process involved deriving the material from waste wood and subjecting it to pyrolysis at 650 °C for 1 h. All chemicals used in this study were of analytical grade and were utilized without further purification. Hydrochloric acid (HCl) and sodium hydroxide (NaOH) were supplied from Merck (Germany). Zirconium(iv) chloride anhydrous (ZrCl_4_, 99.99% purity) was sourced from Sigma-Aldrich (Germany). Diclofenac sodium (DCF, >98.0% purity) and 2-aminoterephthalic acid (C_8_H_7_NO_4_, >98% purity) were obtained from TCI (Japan). N, N-dimethylformamide (DMF, >98% purity), acetonitrile (ACN, maximum 0.001% water), and absolute ethanol were supplied by VWR (UK).

### 2.2. Synthesis of Zr-MOF@C based on UiO-66-NH_2_

A total of 543.44 mg of 2-aminoterephthalic acid and 104.66 mg of carbon (calculated as 10% of the theoretical mass of the MOF precursor) were mixed in 40 mL of DMF and stirred for 20 minutes in a 250 mL screw-cap bottle. A separate solution was prepared by dissolving 503.20 mg of ZrCl_4_ in 20 mL of DMF and 4 mL of 37% HCl, which was subsequently added to the initial mixture. The bottle was sealed, and the solution was stirred at 80 °C for 12 h. Afterward, the reaction mixture was filtered, washed with ACN, and subjected to solvent exchange under autogenous pressure for 2 h using heated ACN at 80 °C. The final product was filtered and dried in an oven at 80 °C for 2 h. This procedure was repeated with varying carbon contents of 20%, 40%, and 60% of the MOF mass.

### 2.3. Characterization

The samples were characterized using advanced analytical techniques at the University of Oulu, Finland. Detailed specifications of each instrument are provided in the SI.

### 2.4. Adsorption studies

A calibration curve was constructed using UV-Visible spectrophotometer to determine the absorbance of DCF solutions at varying concentrations (2.5–20 mg L^−1^) at *λ*_max_ = 276 nm (Fig. S1 in the SI). For adsorption experiments, 0.01 g of each adsorbent was added to 20 mL of 30 mg per L DCF solution at varying pH values (3, 5, 7, and 9), adjusted using 0.1 M HCl and 0.25 M NaOH. The effects of contact time (1–24 h), initial DCF concentrations (25–500 mg L^−1^), temperature (22–55 °C), and adsorbent dose (5, 10, 20, 30, and 40 mg in 20 mL) were systematically studied to evaluate adsorption kinetics, isotherms, and thermodynamics through batch adsorption experiments. The specific experimental conditions used for each adsorption parameter test are summarized in (Table 1). All experiments were performed in duplicate, and average values are reported with corresponding standard deviations. The removal efficiency (%*E*) and adsorption capacity (*q*_e_, mg g^−1^) were calculated using eqn (1) and (2), where *C*_o_ and *C*_e_ (mg L^−1^) represent the initial and equilibrium DCF concentrations, *V* (L) is the solution volume, and *m* (g) is the adsorbent mass:^20^1
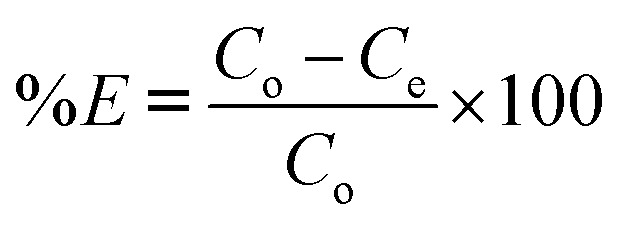
2
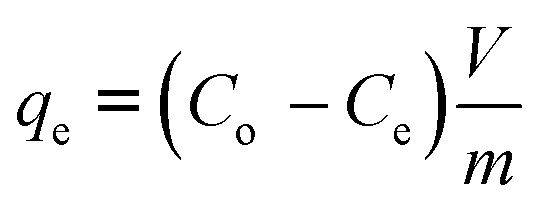


**Table 1:** A summary of the experimental conditions used for each adsorption parameter

Parameter	Effect of pH	Effect of dose	Effect of time	Effect of concentration	Effect of temperature
pH	3–9	10	24	30	22
Adsorbent dose (mg)	7	5–40	24	30	22
Time (h)	7	10	1–24	30	22
Concentration (mg L^−1^)	7	5	24	25–500	22
Temperature (°C)	7	10	5	30	22–55

### 2.5. Desorption and stability tests

The reusability of the adsorbents was examined through desorption and regeneration over four cycles. After DCF adsorption, the adsorbents were washed with 0.1 M HCl, 70% ethanol, and deionized water, then reused for further adsorption cycles. DCF concentrations in the filtrate were measured using UV-Visible spectroscopy after each cycle.

## 3 Results and discussion

### 3.1. Characterization of adsorbent

**Fig. 1:**
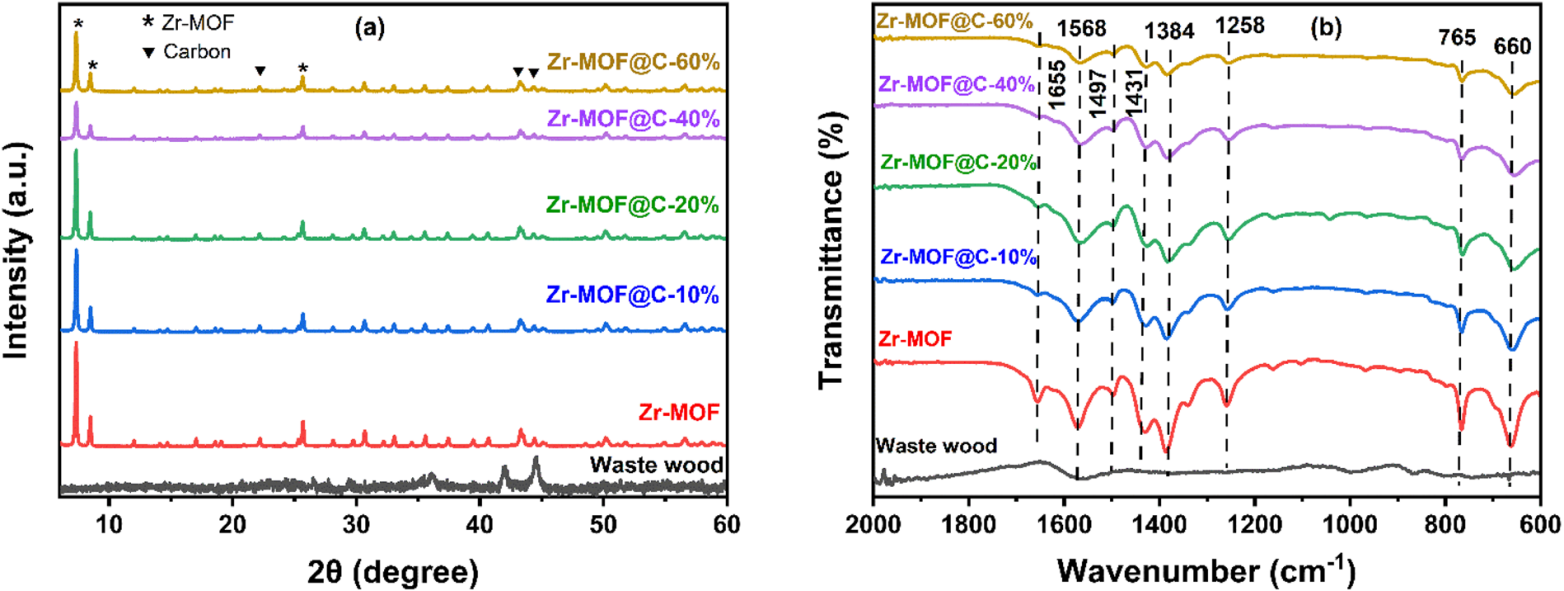
(a) XRD patterns and (b) FTIR Spectra of waste wood (carbon), Zr-MOF, and Zr-MOF@C composites with varying carbon content.

The successful compositing of Zr-MOF on carbon was confirmed through XRD and FTIR spectroscopy. The XRD patterns, as shown in (Fig. 1a), confirmed the successful synthesis of Zr-MOF with characteristic peaks at 2*θ* = 7.3°, 8.5°, and 25.7° (ICDD: 01-085-6809). The waste wood-based carbon sample exhibited characteristic carbon peaks at 2*θ* = 23.4°, 41.9° and 44.3° (ICDD: 04-013-0293). However, since the MOF is crystalline and the carbon is amorphous, it is expected that the sharp MOF peaks dominate the spectrum and mask any broad or weak reflections from the carbon phase in the Zr-MOF@C samples.

The FTIR spectra revealed several characteristic peaks, as shown in (Fig. 1b). The band at 660 cm^−1^ is attributed to μ_3_-O stretching in the Zr_6_O_4_(OH)_4_ clusters,^21^ while the 765 cm^−1^ band corresponds to N–H swinging vibrations.^22^ A peak at 1655 cm^−1^ is assigned to residual DMF from synthesis.^23^ The peaks at 1568 cm^−1^ and 1384 cm^−1^ correspond to the asymmetric and symmetric stretching vibrations of coordinated carboxylate groups, confirming successful ligand coordination.^24^ Additionally, C–C stretching within the aromatic rings appears at 1497 cm^−1^ and 1431 cm^−1^, characteristic of the benzene ring in the 2-amino-terephthalic acid linker.^25^ Finally, the peak at 1258 cm^−1^ is assigned to C–N stretching, further supporting the presence of amine-functionalized ligands.^26^

**Fig. 2:**
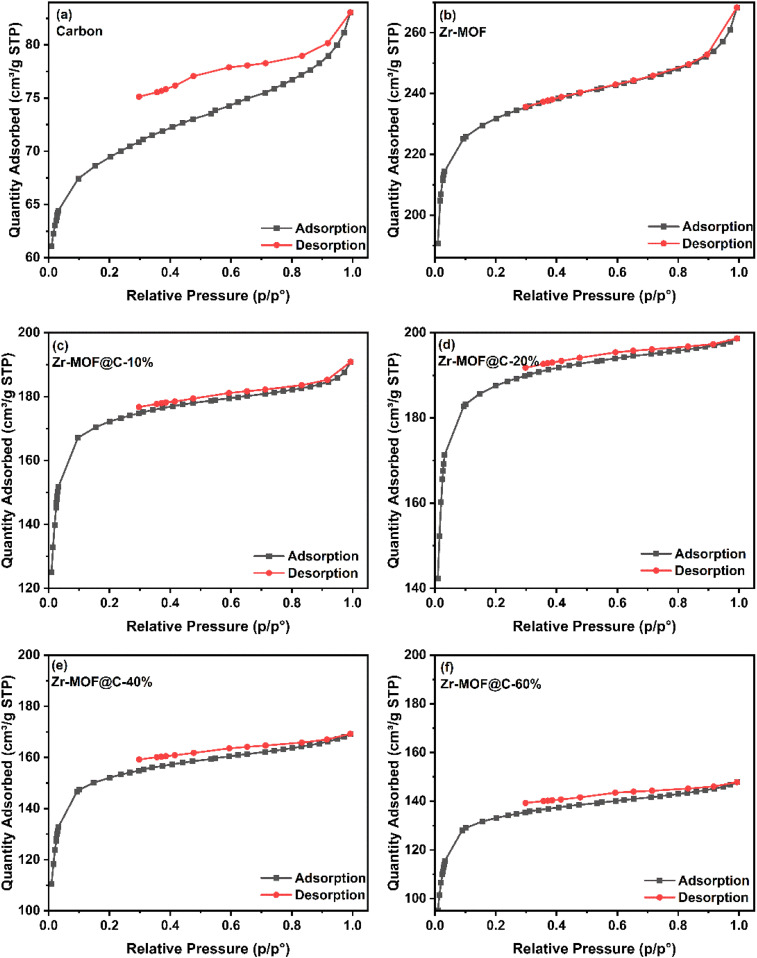
N_2_ adsorption–desorption isotherm plot of (a) carbon, (b) Zr-MOF, and (c–f) Zr-MOF@C composites with varying carbon content.

The BET analysis (Table 2) highlights how carbon incorporation significantly modifies the pore structure of Zr-MOF. While pristine Zr-MOF shows a high surface area of 943 m^2^ g^−1^ and a dominant microporous structure (91.2%) with a pore diameter of 1.7 nm. While adding 10% carbon, the surface area decreases to 672 m^2^ g^−1^, while microporosity slightly increases to 93.2%. A further increase to 20% carbon results in a higher surface area (784 m^2^ g^−1^) and the highest microporosity (98%). These results suggest that carbon addition enhances the microporous character of the composite. However, beyond 20%, higher carbon content led to a decline in surface area and pore volume, likely due to pore blockage. The N_2_ adsorption–desorption isotherms in Fig. 2 exhibit Type IV behaviour with H4-type hysteresis loops, indicating the coexistence of micropores and narrow mesopores.^27^ The sharp uptake at low *P*/*P*_o_ confirms microporosity, while the hysteresis and desorption closure near *P*/*P*_o_ = 0.4 suggest narrow slit-like mesopores formed between MOF crystals and carbon domains.^28–30^ Notably, pristine Zr-MOF shows the highest nitrogen uptake due to its well-developed microporous structure, while the waste wood-derived carbon exhibits much lower uptake. Increasing carbon content from 20% to 60% leads to a gradual decline in N_2_ adsorption, likely due to pore blockage and reduced surface area—consistent with SEM and BET results. Further textural analysis using *t*-plot (Fig. S2) and BJH-derived pore size distribution curves alongside cumulative pore volume plots (Fig S3) in the SI confirmed the dominant microporosity and revealed mesoporous contributions, offering a more complete view of the hierarchical pore structure.

**Table 2:** BET surface area and pore characteristics of the carbon, Zr-MOF and Zr-MOF@C composites

Adsorbents	Surface area (m^2^ g^−1^)	Pore diameter (nm)	Pore volume (cm^3^ g^−1^)	Micro pores (%)	Meso pores (%)	Macro pores (%)
Carbon	275	1.8	0.12	82.5	16.5	1.0
Zr-MOF	943	1.7	0.33	91.2	7.3	1.5
Zr-MOF@C-10%	672	1.7	0.27	93.2	6.0	0.8
Zr-MOF@C-20%	784	1.6	0.27	98.0	1.8	0.2
Zr-MOF@C-40%	593	1.7	0.24	91.7	8.0	0.3
Zr-MOF@C-60%	522	1.7	0.21	92.3	7.4	0.3

**Fig. 3:**
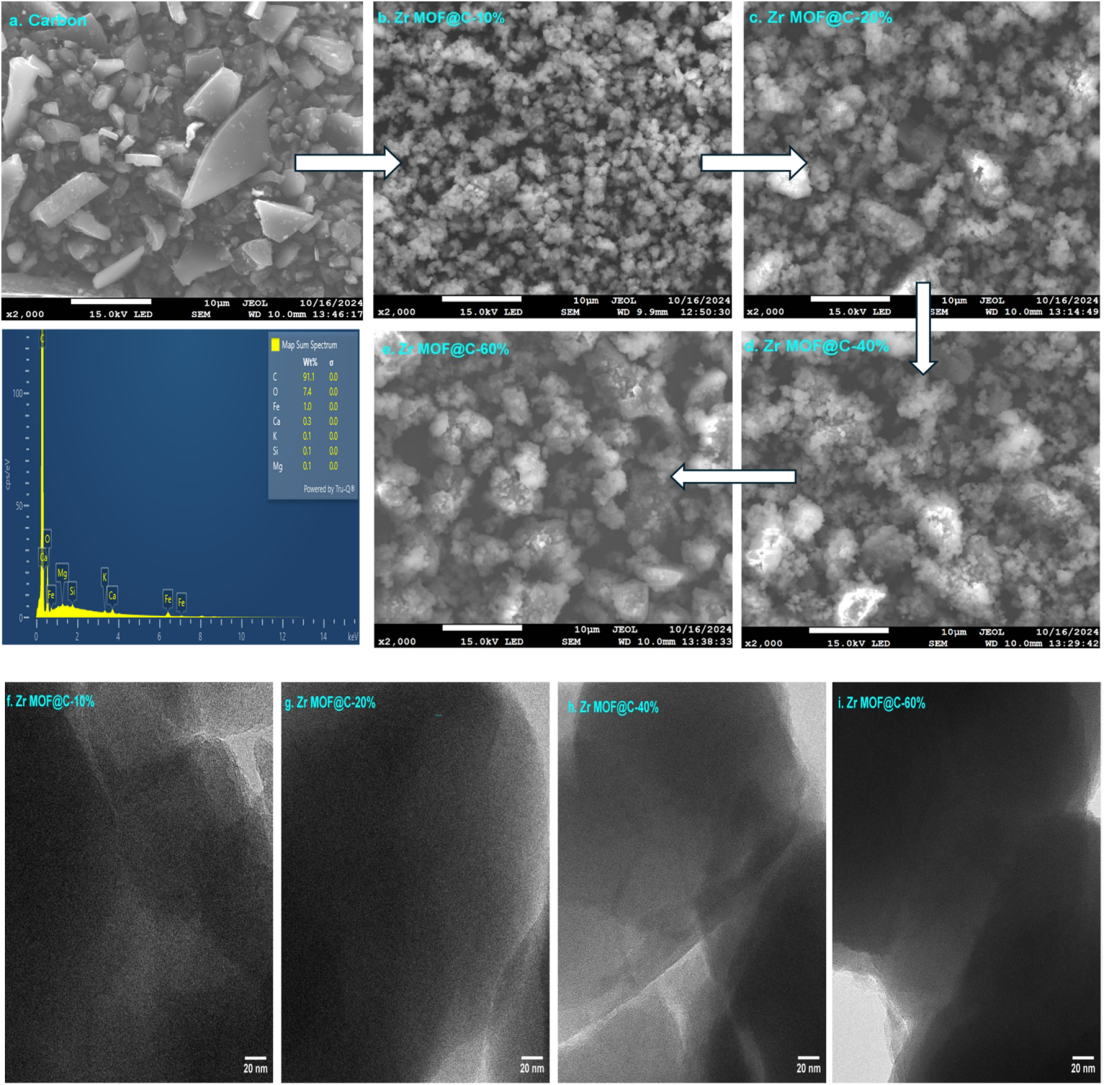
(a–e) SEM images of carbon and Zr-MOF@C composites with varying carbon content, along with the EDX spectrum of waste wood; (f–i) TEM images of Zr-MOF@C composites with varying carbon content.

SEM and TEM were employed to examine the morphological evolution of Zr-MOF composites with varying carbon content (10 wt%, 20 wt%, 40 wt%, and 60 wt%). The SEM image of pure carbon (Fig. 3a) reveals irregular particles with smooth surfaces, typical of carbon. Incorporation of 10% carbon (Fig. 3b) into Zr-MOF shows a uniform coating of carbon particles, partially filling the pores while maintaining structural porosity. At 20% carbon content, both SEM and TEM images (Fig. 3c and g) confirm an optimal balance, with a cohesive and well-distributed carbon coating that enhances compatibility between carbon and Zr-MOF, maintaining pore accessibility and structural stability. As the carbon content increases to 40%, the carbon layer thickens, becoming denser and beginning to obscure the Zr-MOF framework, as seen in both SEM and TEM images (Fig. 3d and h), which suggests a reduction in pore accessibility. At 60% carbon content, the composite is dominated by a thick carbon layer that heavily obscures the Zr-MOF framework, significantly limiting the accessibility of active sites and overall porosity.

To further confirm the structural homogeneity and elemental integration, EDX elemental mapping was performed and is presented in the SI (Fig. S4–S7). These maps demonstrate a uniform distribution of C, Zr, O, and N, indicating successful embedding and homogeneous integration of carbon within the Zr-MOF framework.

### 3.2. Optimizing DCF adsorption on Zr-MOF@C composites: influence of pH, time and initial DCF concentration

**Fig. 4:**
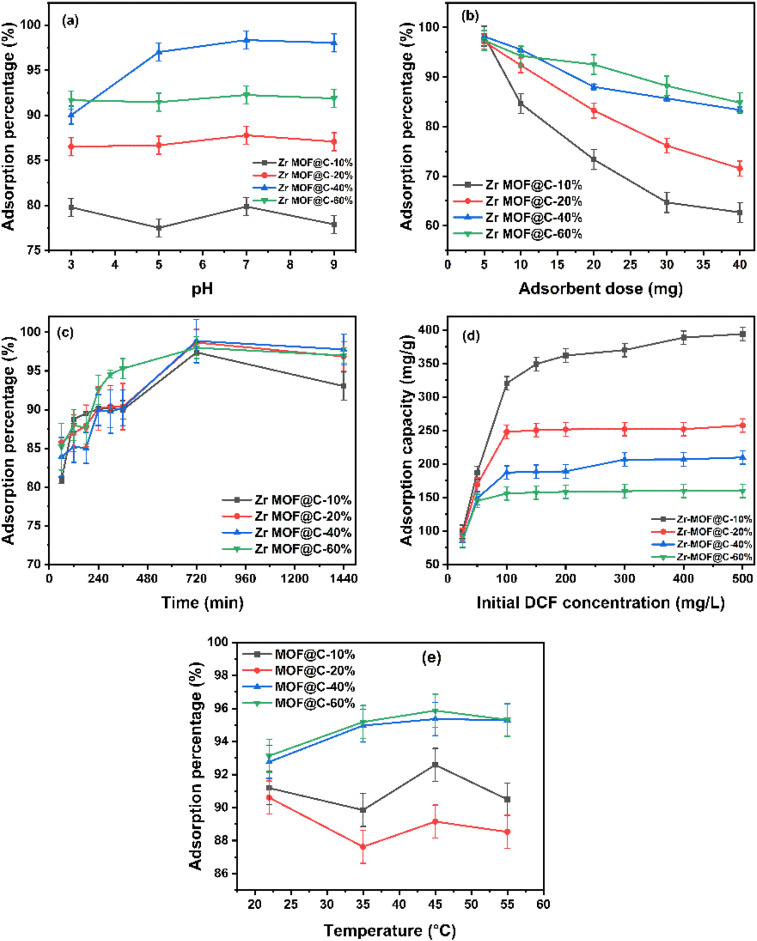
Adsorption performance of Zr-MOF@C composites (a) effect of pH, (b) effect of adsorbent dose, (c) effect of contact time, (d) effect of initial concentration, and (e) effect of temperature.

The adsorption behaviour of Zr-MOF@C composites was examined across a pH range of 3 to 9, with contact durations ranging from 1 to 24 h and initial DCF concentrations varying between 25 mg L^−1^ and 500 mg L^−1^. Based on our previous work, which demonstrated the control of the surface charge of UiO-66-NH_2_ to UiO-66-NH_3_^+^ this study highlights the importance of surface charge modifications.^19^ As shown in (Fig. 4a), neutral pH conditions were found to be optimal for DCF adsorption, attributed to the positively charged surface of the Zr-MOF@C composite after the activation process. The anionic nature of DCF at pH levels above 4 facilitates strong electrostatic interactions with the positively charged composite surface, significantly enhancing adsorption efficiency.^5^ Furthermore, as shown in (Fig. 4b), the adsorption efficiency decreases with increasing adsorbent dose, primarily due to particle aggregation at higher dosages, which reduces the effective surface area and blocks access to micropores.^31^ Regarding contact time, the adsorption percentage increases rapidly during the initial stages of contact, up to 720 minutes, and then reaches equilibrium, as illustrated in Fig. 4c. The rapid adsorption during the initial phase is driven by the abundant availability of active sites on the adsorbent surface, allowing efficient interaction with DCF molecules.^15^ Beyond 720 minutes, the adsorption rate slows as the active sites become saturated, marking the equilibrium phase of the process. Additionally, as shown in Fig. 4d, the adsorption capacity increases with higher DCF concentrations due to the enhanced driving force for mass transfer. At lower DCF concentrations, all samples offer sufficient active sites, resulting in similar adsorption performance. However, at higher concentrations, Zr-MOF@C-10% shows superior uptake, likely due to its balanced surface area and pore structure. In contrast, composites with higher carbon content may experience partial pore blockage or restricted diffusion, limiting their adsorption efficiency under elevated DCF levels. Moreover, as shown in Fig. 4e, the adsorption performance improves with temperature, reaching an optimum at 45 °C. This suggests enhanced DCF diffusion and interaction with the adsorbent. A slight decline at 55 °C may be due to weakened binding or partial desorption.

### 3.3. Kinetics studies

The adsorption kinetics were analyzed using well-established models to investigate the rate and mechanism of adsorption. The pseudo-first order (eqn (3)) and pseudo-second-order (eqn (4)) models were employed to evaluate the adsorption capacity over time, where *q*_*t*_ represents the adsorption capacity at time *t*, and *q*_e_ denotes the equilibrium capacity. The rate constants, *K*_1_ and *K*_2_, describe the adsorption rates for each model, respectively. Furthermore, the Weber–Morris intra-particle diffusion model (eqn (5)) was applied to assess the contribution of diffusion to the adsorption process, where the constant *K*_i_ represents the intra-particle diffusion rate, and *c* accounts for diffusion resistance. The equations are detailed as follows:^32^3
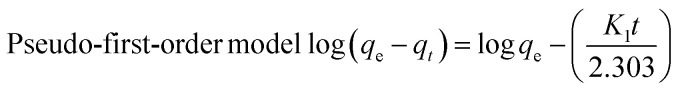
4
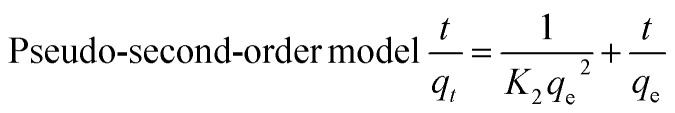

5 Weber Morris intra-particle diffusion model: *q*_*t*_ = *K*_i_*t*^½^ +*c*

**Fig. 5:**
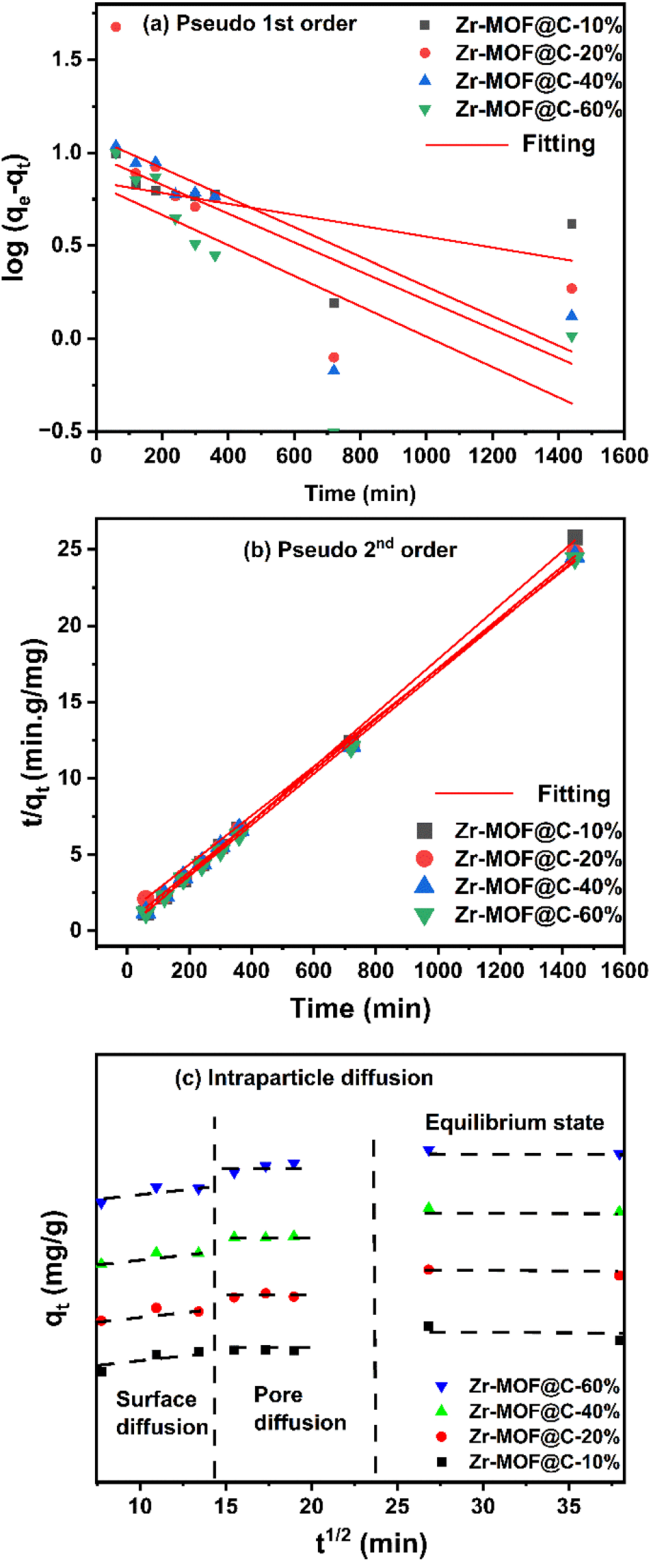
Linear plots for (a) pseudo 1st order; (b) pseudo 2nd order; and (c) intraparticle diffusion kinetics on DCF adsorption. Note: the *y*-axis represents stacked lines plotted with *y*-offsets (origin software).

The pseudo-second-order model provided the best fit for all composites, as evidenced by high correlation coefficients and the close agreement between experimental and calculated adsorption capacities, confirming that chemisorption governs the adsorption process (Table 3). Zr-MOF@C-10% exhibited the fastest adsorption rate, likely due to its surface area and porosity, which enhance the accessibility of active adsorption sites and facilitate the interaction of adsorbate molecules with the adsorbent surface.^33^ The intra-particle diffusion plot (Fig. 5c) reveals a three-stage adsorption process characterized by decreasing rates. The initial stage involves rapid surface adsorption, where the adsorbate diffuses from the solution to the adsorbent surface, which is not considered a rate-limiting step. The second stage corresponds to slower intra-particle diffusion, during which the adsorbate penetrates the adsorbent matrix and diffuses through its pores. The final equilibrium stage exhibits minimal diffusion as the adsorbate concentration diminishes. These results suggest that the adsorption process is multistage, with intra-particle diffusion playing a significant role in influencing the adsorption rate, although it is not the sole rate-determining factor.^34^

**Table 3:** Kinetic parameters for the adsorption of DCF

Adsorbents	*q*_e_ exp (mg g^−1^)	Pseudo 1st order			Pseudo 2nd order		
		*q*_e_ cal (mg g^−1^)	*K*_1_ (min^−1^)	*R* ^2^	*q*_e_ cal (mg g^−1^)	*K*_2_ (g mg^−1^ min^−1^)	*R* ^2^
Zr-MOF@C-10%	55.85	2.32	0.69 × 10^3^	0.69	56.49	23.33 × 10^−3^	0.999
Zr-MOF@C-20%	58.14	2.94	1.84 × 10^3^	0.49	59.88	6.68 × 10^−3^	0.998
Zr-MOF@C-40%	58.68	2.67	1.84 × 10^3^	0.66	59.88	13.59 × 10^−3^	0.999
Zr-MOF@C-60%	58.96	2.29	0.46 × 10^3^	0.55	59.88	8.83 × 10^−3^	0.999

### 3.4. Adsorption isotherms

The equilibrium adsorption data was analyzed using two widely recognized models to evaluate the interactions between the adsorbate and the adsorbent. The Langmuir isotherm (eqn (6)) assumes monolayer adsorption on a homogeneous surface and is suitable for chemisorption processes. In this model, the adsorption capacity (*q*_e_) at equilibrium is related to the maximum adsorption capacity (*q*_m_) and the Langmuir constant (*b*), which were determined through linear plots of *C*_e_/*q*_e_ against *C*_e_.6
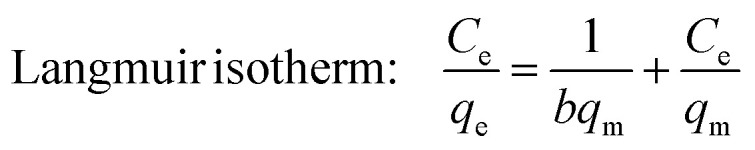


The Freundlich isotherm (eqn (7)), on the other hand, assumes multilayer adsorption on a heterogeneous surface, with the adsorbent's surface energy decreasing as adsorption progresses. The Freundlich constants (*K*_f_) and the heterogeneity parameter (1/*n*) were determined from linear plots of log(*q*_e_) against log(*C*_e_).7
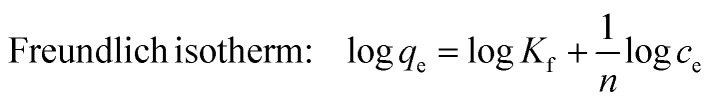


**Fig. 6:**
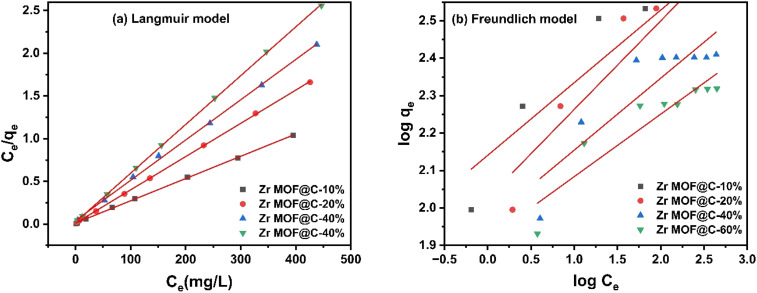
Adsorption isothermal models for DCF adsorption (a) Langmuir isotherm; (b) Freundlich isotherm.

The adsorption equilibrium data were best described by the Langmuir isotherm model, as evidenced by the high *R*^2^ = 0.999 values for all composites (Fig. 6a). This indicates that adsorption occurs as a monolayer on a homogeneous surface. The maximum adsorption capacity (*q*_m_) decreased with increasing carbon content (Table 4), with Zr MOF@C-10% exhibiting the highest *q*_m_ (384.6 mg g^−1^) and Zr MOF@C-60% showing the lowest (175.4 mg g^−1^). This reduction in *q*_m_ is likely due to excessive carbon coverage at higher carbon contents, which reduces the accessibility of active adsorption sites on the Zr-MOF framework.

**Table 4:** Langmuir and Freundlich isotherms parameters

Adsorbent	Langmuir model			Freundlich model		
	*q*_m_ (mg g^−1^)	*b* (L mg^−1^)	*R* ^2^	*K*_f_ [mg g^−1^ (mg L^−1^)^*n*^]	1/*n*	*R* ^2^
Zr-MOF@C-10%	384.6	0.217	0.999	8.5	5.2	0.882
Zr-MOF@C-20%	256.4	0.355	0.999	7.6	6.0	0.799
Zr-MOF@C-40%	212.8	0.116	0.999	6.8	6.0	0.832
Zr-MOF@C-60%	175.4	0.238	0.999	7.0	8.1	0.760

The Langmuir constant (*b*), which reflects the adsorption affinity, was highest for Zr-MOF@C-20% (0.355 L mg^−1^), suggesting stronger adsorbate–adsorbent interactions at this composition.^35^ This indicates that while Zr-MOF@C-10% achieved the highest adsorption capacity, Zr-MOF@C-20% demonstrated a superior balance between adsorption capacity and affinity, making it potentially more efficient under practical conditions. Notably, the maximum adsorption capacities (*q*_m_) for this work stand at almost 385 mg g^−1^, exceeding most adsorption capacities reported for other adsorbents, as summarized in Table 5.

**Table 5:** Maximum adsorption capacities of Zr-MOFs and other adsorbents for the aqueous phase adsorption of DCF

Adsorbents	Dosage/DCF solution volume	*T* (°C)	pH	*q*_m_ (mg g^−1^)	References
Activated carbon	15 mg/50 mL	25 °C	Neutral	147	36
Acid treated zeolite	20 mg/50 mL	20 °C	6.0	86	37
PCDM-1000	4 mg/50 mL	25 °C	5.5	320	38
MOF-303	3 mg/10 mL	25 °C	7.0	335	39
NH_2_-MIL-53(Fe)/CS	5 mg/50 mL	25 °C	—	728	40
d-MOF-801(35)	5 mg/10 mL	25 °C	7.0	680	41
PTA @MIL101(Cr)	5 mg/10 mL	25 °C	5.5	413	42
UiO-66-NH_2_ (25)	3 mg/15 mL	25 °C	5.6	357	43
18% SO_3_H-UiO-66	5 mg/50 mL	25 °C	5.4	263	15
UiO-66-(COOH)_2_	20 mg/10 mL	25 °C	7.0	481	44
Fe_3_O_4_-FeBTC	3 mg/50 mL	30 °C	4.5	347	45
Zr-MOF@C-10%	5 mg/20 mL	22 °C	7.0	385	This study

### 3.5. Thermodynamics analysis

The thermodynamic behaviour of the adsorption process was evaluated for the optimum sample Zr-MOF@C-10%, by determining the enthalpy (Δ*H*°), entropy (Δ*S*°), and Gibbs energy (Δ*G*°) changes to assess spontaneity and temperature dependence. The distribution coefficient (*K*_d_) was calculated as 
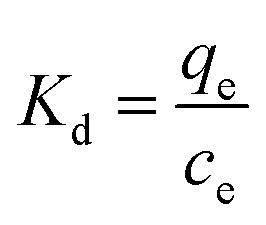
, and the thermodynamic parameters were derived using the van't Hoff equation, and the corresponding plot is provided in Fig. S8 (SI).8
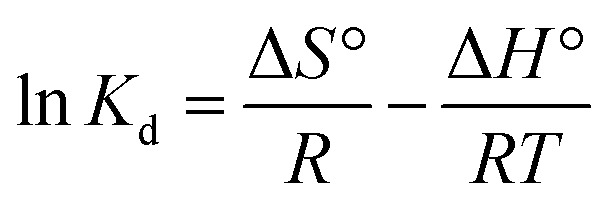

9 Δ*G*° = −*RT* ln *K*_d_

The thermodynamic parameters for Zr-MOF@C-10% indicated an endothermic and spontaneous adsorption process. As shown in (Table 6), the positive Δ*H*° suggests that the adsorption efficiency improves with increasing temperature. The positive Δ*S*° reflects increased randomness at the solid–liquid interface, which enhances the interaction between the adsorbent and DCF molecules. The negative Δ*G*° values at all temperatures confirm the spontaneity of the process and further suggest the coexistence of both physisorption and chemisorption, in agreement with the proposed adsorption mechanism.^46,47^

**Table 6:** The thermodynamic parameters for DCF adsorption

Adsorbent	Δ*H*° (kJ mol^−1^)	Δ*S*° (J mol^−1^ K^−1^)	Δ*G*° (kJ mol^−1^) at different temperatures			
			295 K	308 K	318 K	328 K
Zr-MOF@C-10%	3.36	35.61	−7.15	−7.36	−8.51	−8.03

### 3.6. Proposed mechanism for the adsorption of DCF

**Fig. 7:**
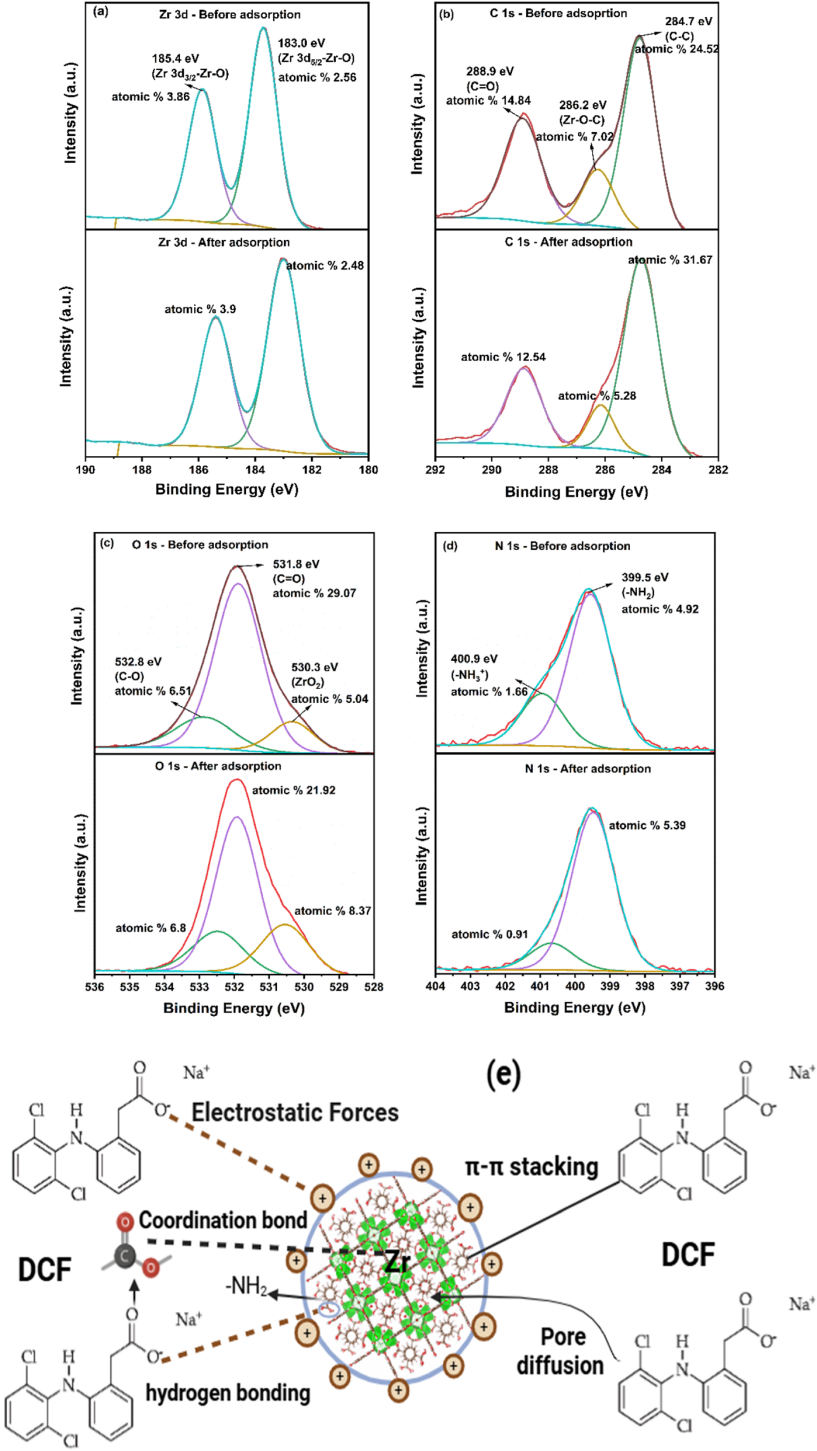
(a–d) XPS analysis showing the changes in the surface composition of Zr-MOF@C after DCF adsorption; (e) proposed mechanism of the adsorption of DCF on Zr MOF@C.

The adsorption of DCF onto Zr-MOF@C is driven by a combination of chemical and physical interactions as illustrated in (Fig. 7e). The positively charged amino groups (–NH_2_) on the framework attract the negatively charged carboxylate groups of DCF, enhancing adsorption efficiency through strong electrostatic forces.^15^ Additionally, the –NH_2_ groups form hydrogen bonds with the carboxyl functional groups of DCF, which play a critical role in stabilizing the adsorption process.^44^ Furthermore, π–π stacking interactions occur between the aromatic rings of the Zr MOF@C framework and the DCF molecules, further stabilizing the adsorbed state.^48^ These interactions, along with the framework's porous structure, significantly improve the composite's adsorption capacity.^49^ Additionally, the carboxylate group of DCF coordinates with the Zr metal centers, indicating the presence of specific binding interactions characteristic of chemisorption.^50^ FTIR analysis (Fig. S9a) supports these findings, showing distinct shifts and intensity changes after DCF adsorption, particularly at ∼1600 cm^−1^ and 1400 cm^−1^, which correspond to the aromatic and carboxylate vibrations of DCF.^51^ A noticeable broadening in the 3200–3600 cm^−1^ region was also observed, suggesting potential changes in surface hydroxyl or amine vibrations involved in hydrogen bonding. These spectral variations confirm the presence of π–π stacking, electrostatic interactions, and hydrogen bonding mechanisms contributing to adsorption.

XPS analysis revealed notable changes in the surface composition of Zr-MOF@C after DCF adsorption as shown in (Fig. 7a–d), including significant shifts in the oxygen (O 1s), nitrogen (N 1s), and carbon (C 1s) peaks, confirming the adsorption of DCF. The C 1s spectra showed an increase in aromatic C–C content from 24.5% to 37.7%, suggesting enhanced π–π stacking interactions *via* the carbon domains. Notably, the emergence of a peak at 286.2 eV in the C 1s spectrum, assigned to C–O–Zr bonding, indicates potential interfacial interactions between the carbon matrix and Zr-MOF framework.^52^ Additional details of the XPS analysis, including surface atomic compositions before and after DCF adsorption, are provided in the SI (Table S1 and Fig. S10).

### 3.7. Regeneration performance and stability of Zr-MOF@C

**Fig. 8:**
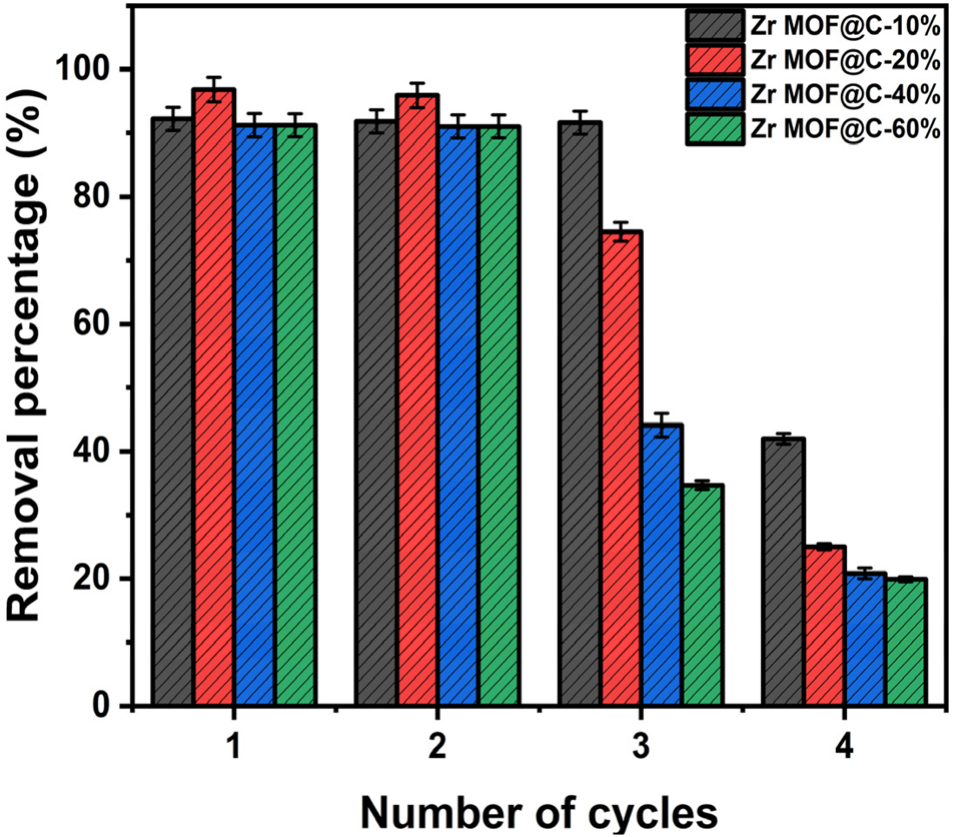
Removal efficiency of Zr-MOF@C composites across multiple adsorption–desorption cycles (at 22 °C, time 24 h; pH 7; dose 10 mg; and initial concentration 30 mg L^−1^).

The reusability of the composites was evaluated over four adsorption–desorption cycles, as shown in (Fig. 8). All materials maintained high removal efficiencies (>90%) during the first two cycles, demonstrating effective regeneration. However, efficiencies declined in the third and fourth cycles, likely due to pore blockage, reducing available active sites for adsorption.^53^ Among the composites, Zr-MOF@C-10% exhibited the most stable performance, attributed to the high structural stability of the Zr MOF framework under repeated cycles. Stability tests were conducted under acidic (pH 2), neutral (pH 7), and alkaline (pH 12) conditions. Zr leaching remained minimal across all pH levels, with no noticeable increase under any condition. XPS analysis (Fig. S10) showed no significant structural changes before and after adsorption. Additionally, XRD patterns (Fig. S9b) confirmed the retention of the characteristic peaks of Zr MOF at 2*θ* = 7.3°, 8.5°, and 25.7°, indicating that the MOF framework remained intact, confirming the excellent stability of the adsorbent under varying environmental conditions.

## 4 Conclusions

This study demonstrates the potential of UiO-66-NH_2_-based Zr-MOF@C composites, synthesized with varying contents of biomass-derived carbon, for the effective removal of diclofenac sodium (DCF) from aqueous solutions. Structural characterization confirmed successful composite formation, and adsorption studies identified Zr-MOF@C-10% as the most efficient material, achieving a maximum capacity of 385 mg g^−1^. This performance is attributed to its optimal balance of surface area, microporosity, and accessible active sites. Regeneration tests showed high removal efficiency (>90%) in the first two cycles, with Zr-MOF@C-10% exhibiting the best stability across repeated use. The adsorption process followed pseudo-second-order kinetics and Langmuir isotherm behavior, indicating monolayer chemisorption driven by electrostatic interactions, hydrogen bonding, and π–π stacking. Thermodynamic analysis confirmed the spontaneity of the process. Overall, this work presents a sustainable, low-cost, and reusable adsorbent material, offering a promising strategy for pharmaceutical pollutant remediation in water treatment applications.

## Author contributions

Sherif Hegazy: conceptualization, methodology, formal analysis, investigation, data analysis and writing – original draft. Konsta Saaranen: methodology. Tao Hu: formal analysis. Sari Tuomikoski: supervision and writing – review & editing. Ulla Lassi: supervision and writing – review & editing, resources, funding acquisition. Varsha Srivastava: supervision and writing – review & editing.

## Conflicts of interest

The authors declare no conflict of interest.

## Supplementary Material

RA-015-D5RA04089B-s001

## Data Availability

The data presented in this study is available on request from the corresponding author. All data generated or analyzed during this study are included in this manuscript and supplementary information (SI). Supplementary information is available. See DOI: https://doi.org/10.1039/d5ra04089b.
