# Supplementary material for: Impact of carbon content on the adsorptive performance of Zr-MOF composites for diclofenac sodium removal
Source: RSC Adv. 2025 Oct 27;15(48):41048–60. doi: 10.1039/d5ra04089b (PMC12557715; doi:10.1039/d5ra04089b)
Supplement: RA-015-D5RA04089B-s001 [file RA-015-D5RA04089B-s001.pdf]

Supplementary Information (SI) for RSC Advances.
This journal is © The Royal Society of Chemistry 2025

Supporting Information

Impact of Carbon Content on the Adsorptive Performance of Zr-MOF Composites for Diclofenac Sodium Removal

Sherif Hegazy[a], Konsta Saaranen[a], Tao Hu[a], Sari Tuomikoski[a], Ulla Lassi[a,b], Varsha Srivastava[a*]

[a]Research Unit of Sustainable Chemistry, University of Oulu, P.O. Box 4300, FI-90014 Oulu, Finland

[b]Kokkola University Consortium Chydenius, University of Jyväskylä, FI-67100 Kokkola, Finland

***Corresponding Author:** Tel: +358414712348, **varsha.srivastava@oulu.fi**

S1: Material characterization

The crystalline structure of the synthesized samples was analysed using X-ray diffraction (XRD) on a PANalytical X'Pert Pro diffractometer (Almelo, Netherlands), equipped with monochromatic Cu $K\alpha_1$ radiation (λ = 1.5406 Å). Measurements were carried out at 45 kV and 40 mA over a 2θ range of 6°–60°, with a step size of 0.017°. The reference patterns for the parent MOF and carbon samples were obtained from the ICDD (International Centre for Diffraction Data) PDF-4+2024 database. Fourier-transform infrared (FTIR) spectroscopy was performed using a Perkin Elmer Spectrum One ATR-FTIR spectrometer (USA), operating in the range of 400–4000 cm^{-1} with a resolution of 4 cm^{-1} and a scan rate of 500 scans/min, to identify functional groups and interaction changes before and after adsorption.

Surface chemical composition and oxidation states were analyzed by X-ray photoelectron spectroscopy (XPS) using a Thermo Fisher Scientific ESCALAB 250Xi system (Waltham, USA) with monochromatic Al Kα radiation (1486.7 eV) and a 900 μm spot size. Survey and high-resolution spectra were collected at pass energies of 150 eV and 20 eV, respectively. Powder samples were

mounted on a gold sample holder, and charge compensation was applied using the C 1s peak at 284.8 eV as reference. Spectral analysis and peak fitting were performed using the Avantage V5 software with a smart background and the TXFN fitting model in accordance with internal "XPS Knowledge" protocols. Specific surface area and porosity were evaluated using nitrogen adsorption–desorption isotherms at 77.3 K, obtained via a Micromeritics 3 Flex instrument (Micromeritics Instruments, Norcross, GA, USA). Samples were first degassed at 140 °C for 3 h under a vacuum pressure of 2.7 mbar to eliminate adsorbed gases and moisture. Isotherms were collected under isothermal conditions by dosing nitrogen incrementally. BET surface area (SSA) of samples were calculated using the Brunauer–Emmett–Teller (BET) method [1], while total pore volume was determined using non-local density functional theory (NLDFT)[2], assuming a slit-pore geometry. A sample mass of 0.1617 g was used with a free space calibration of 57.4323 cm^3 and an equilibration interval of 5 s.

The morphology of the samples was examined using a Zeiss Sigma field-emission scanning electron microscope (FESEM, Jena, Germany) at an accelerating voltage of 5 kV, across magnifications from 150× to 100,000×. High-resolution transmission electron microscopy (HR-TEM) and energy-filtered TEM (EFTEM/STEM) were performed using a JEOL JEM-2200FS (Akishima, Japan) at 200 kV. Samples were prepared by ultrasonically dispersing the powder in ethanol and drop-casting onto carbon-coated copper grids (Lacey F/C, 200 mesh Cu, TED Pella Inc.). UV–Visible spectrophotometry was performed using a SHIMADZU UV-1800 instrument (Shimadzu Corporation, Kyoto, Japan) to determine DCF concentrations by measuring absorbance at λ_max = 276 nm.

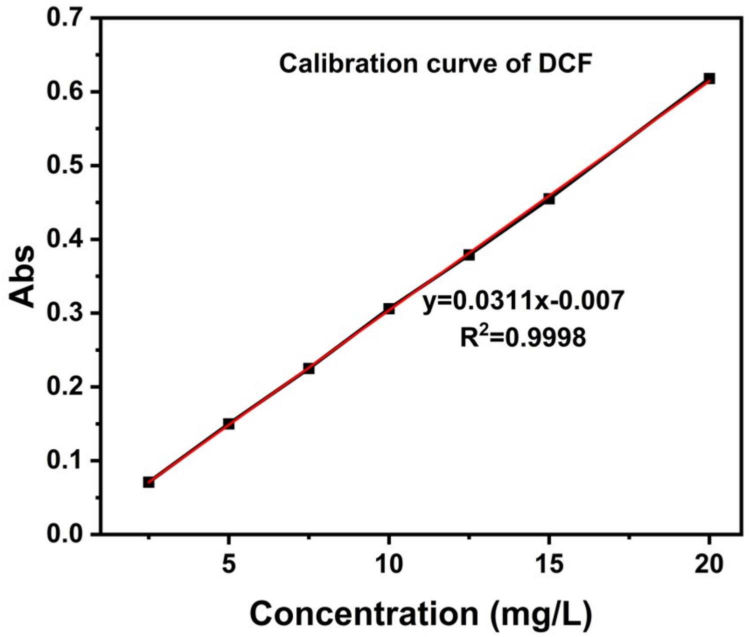

Fig.S1: Calibration curve of DCF.

Additional surface textural analysis:

To further validate the microporous nature of the synthesized materials, **Fig. S2** presents the *t-plot analysis* based on the Harkins and Jura thickness equation:

$$t\ (nm) = \sqrt{\frac{0.1399}{0.034 - \log\left(\frac{P}{P_0}\right)}}$$

This method enables the estimation of micropore area, external surface area and micropore volume through linear fitting in the statistical thickness range (indicated in red in each graph). The results confirm that the parent Zr-MOF exhibits the highest micropore area (825 m²/g), which systematically decreases as the carbon content increases in the composites. This trend supports the interpretation that carbon partially blocks micropores within the MOF structure, aligning with the surface area and pore volume data reported in **Table 2** of the main manuscript.

In addition, **Fig. S3** provides BJH-derived pore size distribution curves alongside cumulative pore volume plots for all materials. While the BJH method is less suitable for accurately quantifying micropores (<2 nm), it is included to give a full view on the hierarchical porosity of the carbon networks. This porosity evolution upon carbon incorporation is crucial for improving molecular diffusion and accessibility to active sites during adsorption.[3]

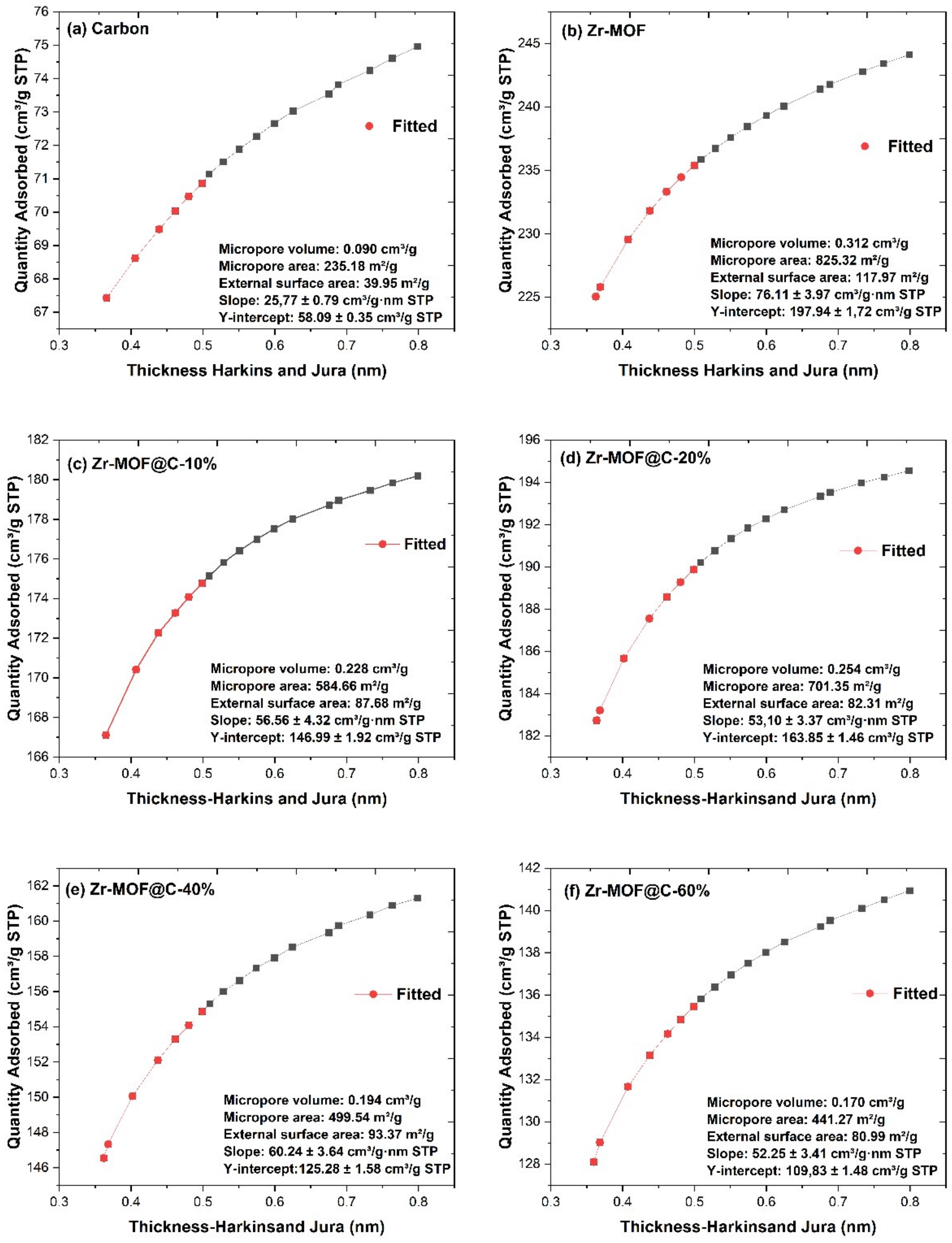

Fig. S2. t-Plot analysis of N_2 adsorption isotherms using the Harkins and Jura model for (a) Carbon, (b) Zr-MOF, (c) Zr-MOF@C-10%, (d) Zr-MOF@C-20%, (e) Zr-MOF@C-40%, and (f) Zr-MOF@C-60%. Micropore area, external surface area and micropore volume were estimated from the linear fitting region.

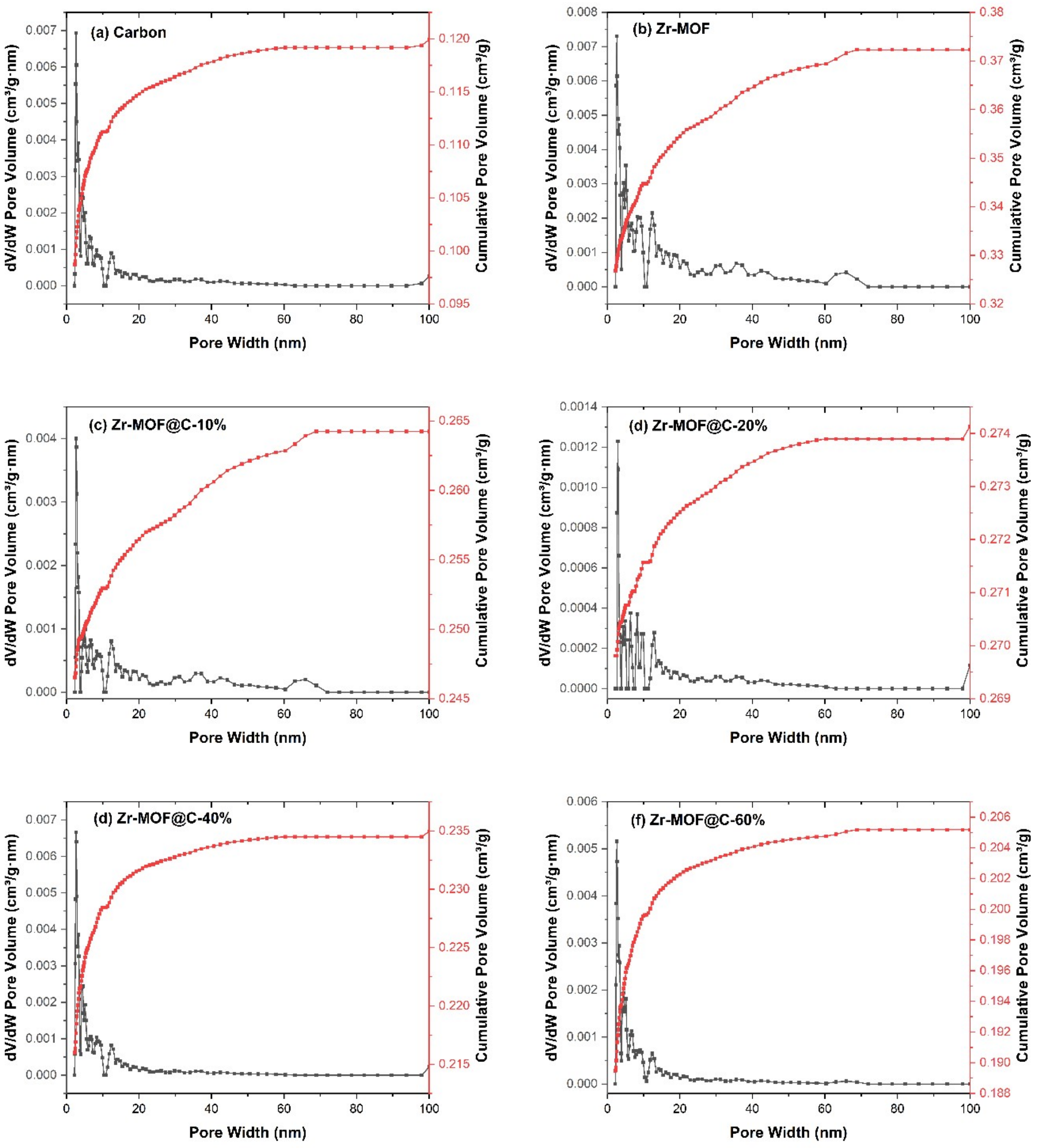

Fig. S3. BJH pore size distribution and cumulative pore volume plots for (a) Carbon, (b) Zr-MOF, (c) Zr-MOF@C-10%, (d) Zr-MOF@C-20%, (e) Zr-MOF@C-40%, and (f) Zr-MOF@C-60%.

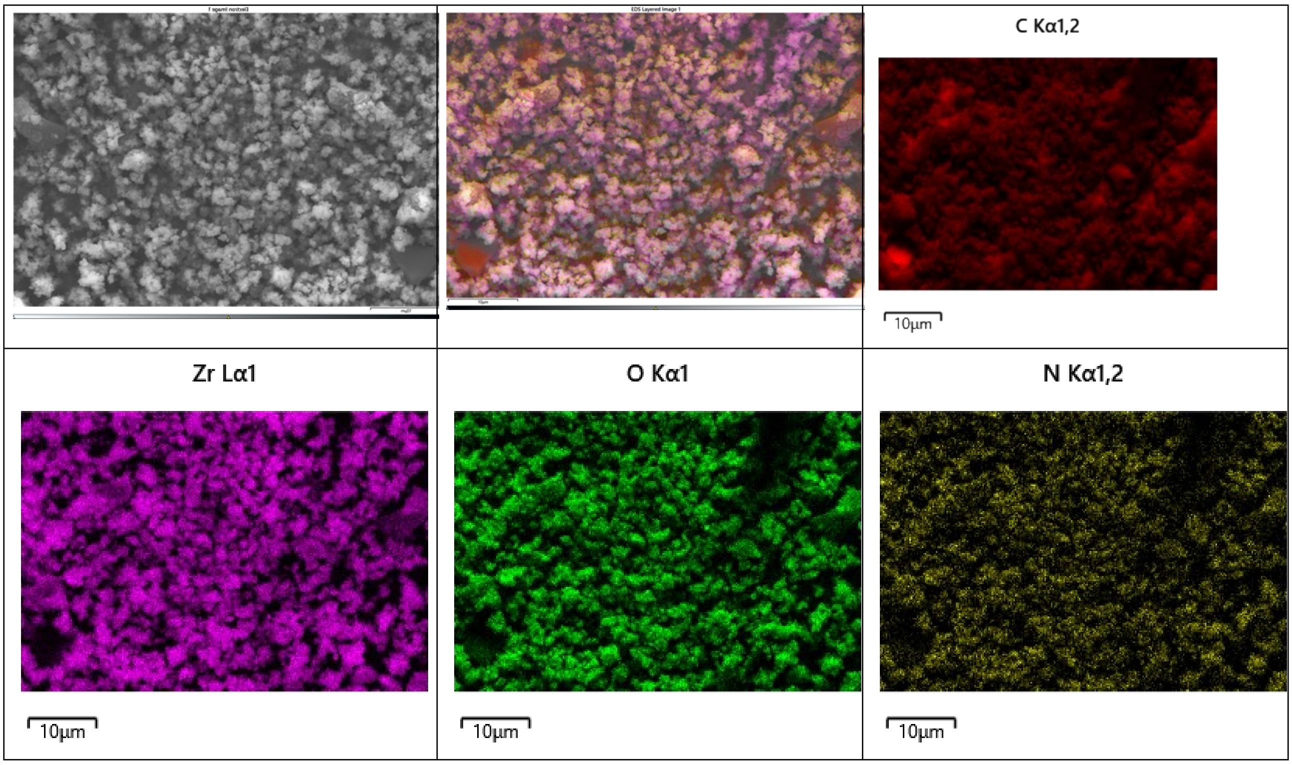

Fig S4: EDX mapping images of Zr-MOF-10%.

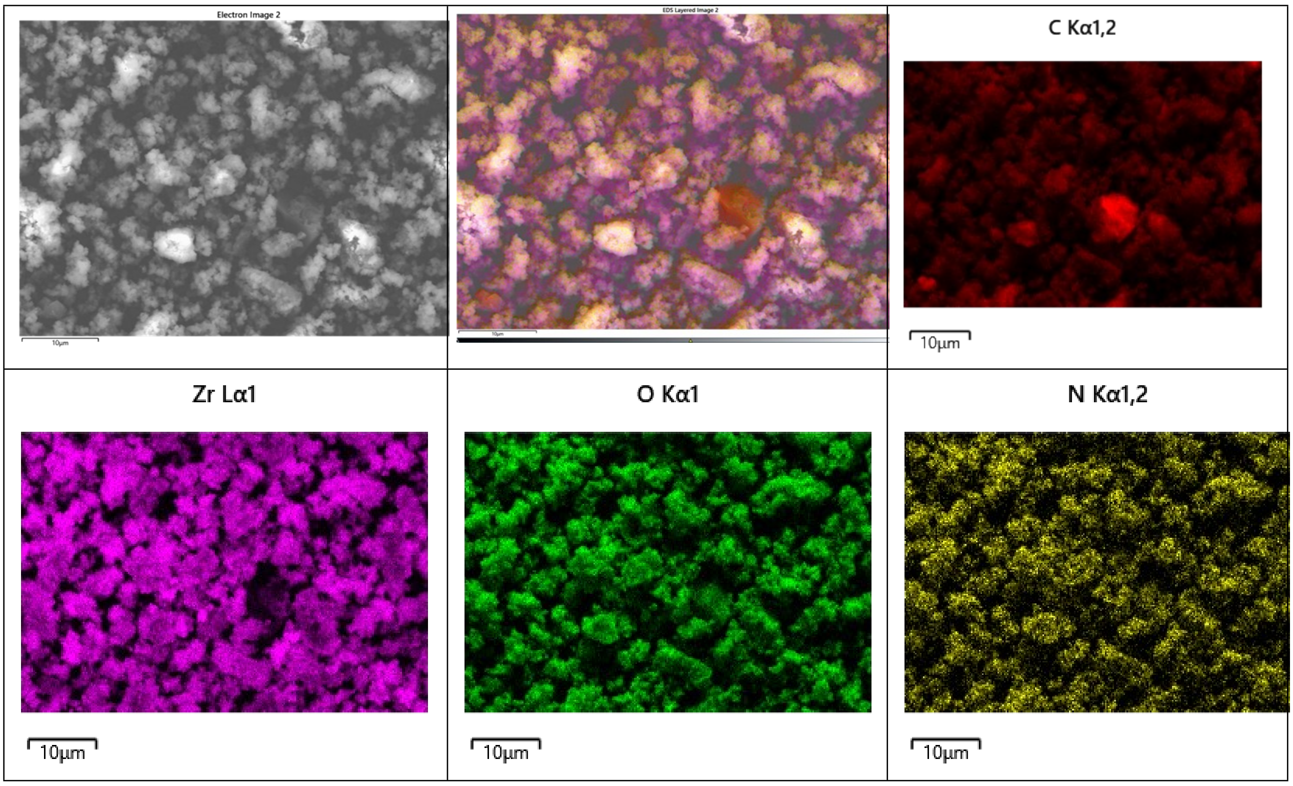

Fig S5: EDX mapping images of Zr-MOF-20%.

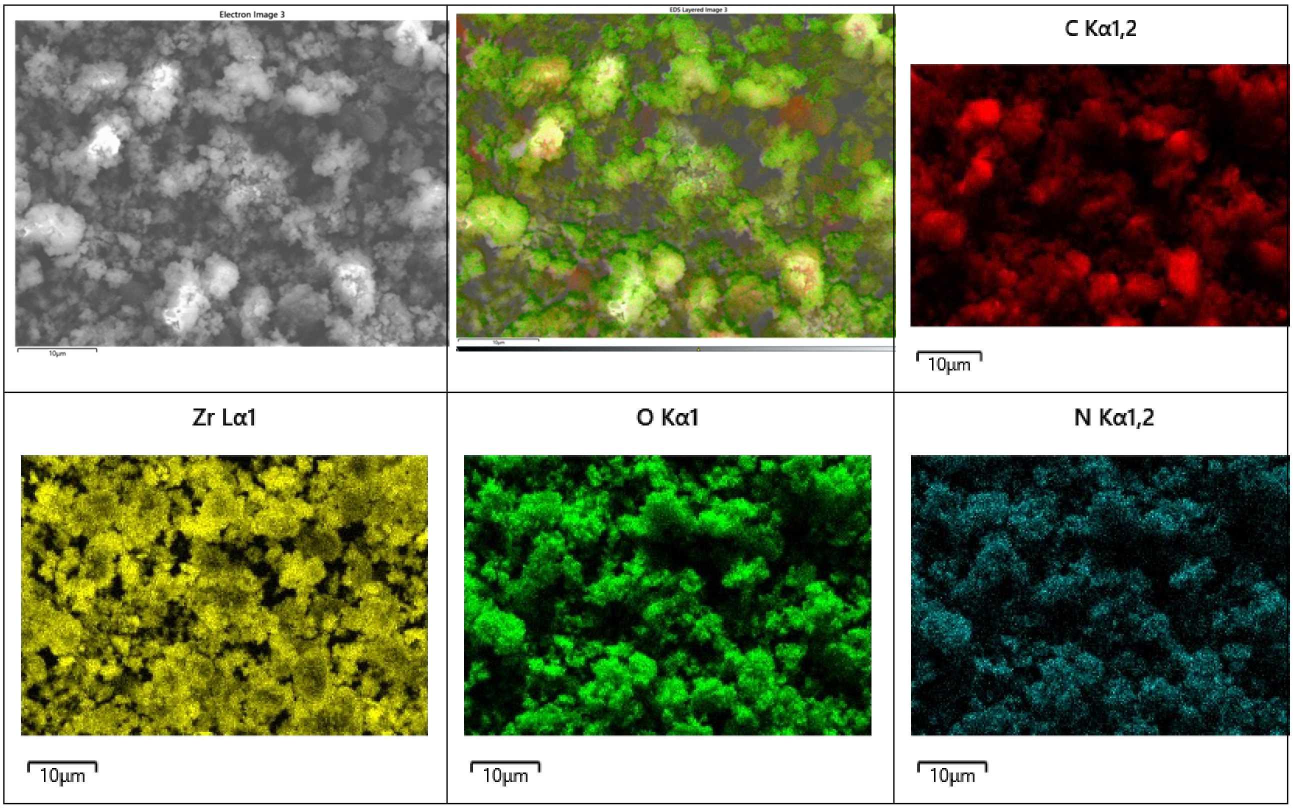

Fig. S6: EDX mapping images of Zr-MOF-40%.

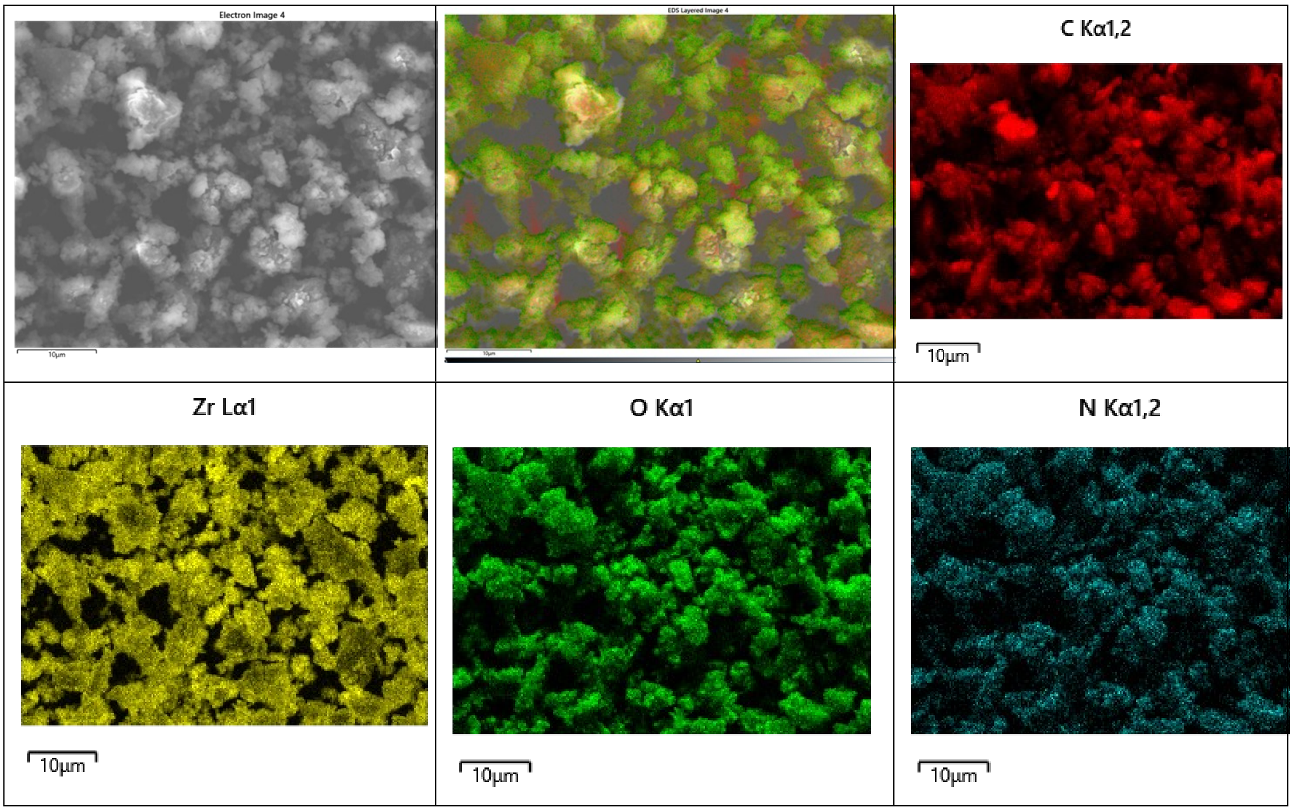

Fig. S7: EDX mapping images of Zr-MOF-60%.

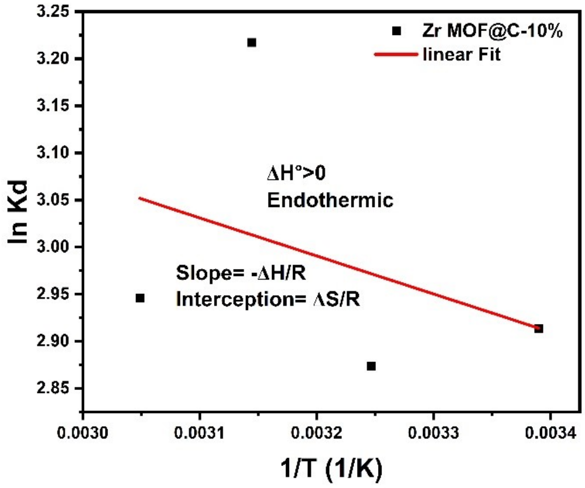

Fig. S8. Van't Hoff plot for determining thermodynamic parameters of DCF adsorption onto Zr-MOF@C-10%. (The linear fit was used to calculate the thermodynamic parameters (ΔH° and ΔS°) reported in Section 3.5 of the main manuscript. Although the fit shows some deviation from linearity, it still provides a reasonable estimate of the thermodynamic parameters)[4,5].

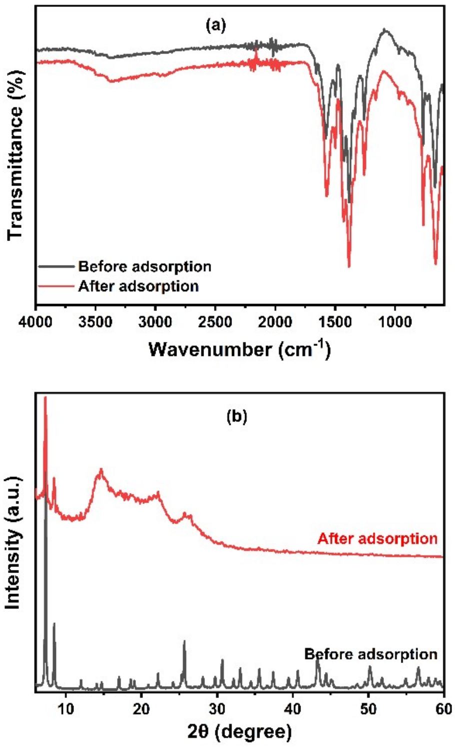

Fig. S9: (a) FTIR spectra and **(b)** XRD patterns of Zr-MOF@C-10% composite before and after DCF adsorption (without any washing steps)

XPS analysis revealed notable changes in the surface composition of Zr-MOF@C composites before and after DCF adsorption, as shown in **(Fig.7d).** The nitrogen (N1s) atomic percentage decreased from 1.66% to 0.91%, suggesting interactions between the surface nitrogen-containing groups and DCF molecules, while a slight increase in the atomic percentage of other nitrogen species was observed. Zirconium (Zr3d) remained stable, with a negligible change in atomic percentages (Fig. 7a), indicating the structural integrity of the Zr-MOF framework during adsorption. The oxygen (O1s) peaks revealed significant changes **(Fig. 7c),** with a reduction in atomic percentage from 29.07% to 21.92%, accompanied by an increase in oxygen species associated with adsorbed DCF molecules. This indicates the active involvement of oxygen-containing functional groups, likely through hydrogen bonding or electrostatic interactions[6,7]. Carbon (C1s) exhibited notable changes **(Fig. 7b),** with the atomic percentage increasing from 24.52% to 31.67%, confirming the adsorption of DCF molecules, which contributed additional carbon species to the surface[8] . Finally, the appearance of a new Cl $2p_{3/2}$ peak at 200.5 eV **(Fig. S10)** with an atomic percentage of 0.73% confirmed the successful adsorption of DCF, as chlorine is a characteristic element of its molecular structure.

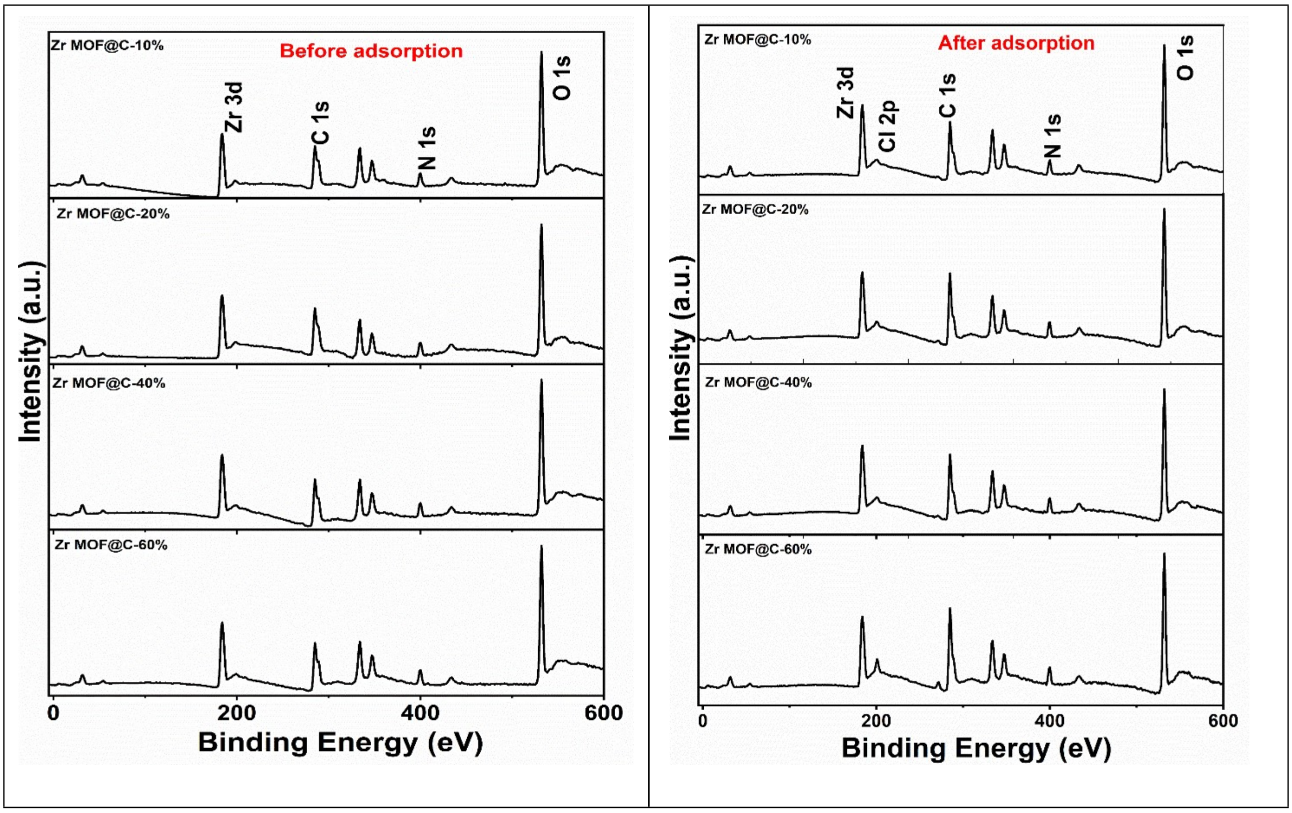

Fig. S10: XPS spectra of Zr-MOF@C composites before and after adsorption.

Table S1. Surface Atomic Composition (Atom%) and High-Resolution XPS Peak Assignments for Zr-MOF@C Composites Before and After DCF Adsorption.

**Zr-MOF@C composites samples**		**C1s**			**O1s**			**N1s**		**Zr3d5/2**
		C-C 284.8 eV	C-O-Zr 286.2 eV	C=O 288.9 eV	Zr-O 530.3 eV	C=O 532.9 eV	C-O 532.9 eV	NH_2 399.5 eV	NH_3 400.9 eV	Zr-O 183.0 eV
**Before adsorption**	Zr MOF@C-10%	24.5	7.0	14.8	5	29.1	6.5	4.9	1.7	2.6
	Zr MOF@C-20%	25.9	7.4	14.8	4.9	29.5	4.5	5.1	1.9	2.4
	Zr MOF@C-40%	26.4	6.2	14.7	7.4	23	9.4	5.2	1.4	2.5
	Zr MOF@C-60%	27.2	5.6	14.3	7.4	26.8	5.4	5.4	1.2	2.5
**After adsorption**	Zr MOF@C-10%	31.7	5.3	12.5	8.4	21.9	6.8	5.4	0.9	2.5
	Zr MOF@C-20%	34.9	6.2	10.7	5.7	25.9	5.7	5.2	0.8	2.2
	Zr MOF@C-40%	35.1	6.0	11.7	7.8	23.3	3.8	4.7	1.2	2.4
	Zr MOF@C-60%	36.2	8.5	10.3	7.8	14.2	10.2	5.2	1.1	2.1

References

1) S. Brunauer, P. H. Emmett and E. Teller, *J Am Chem Soc*, DOI:10.1021/ja01269a023.
2) C. Lastoskie, K. E. Gubbins and N. Quirke, *Langmuir*, DOI:10.1021/la00034a032.
3) P. Deng, S. Lei, W. Wang, W. Zhou, X. Ou, L. Chen, Y. Xiao and B. Cheng, *J Mater Sci*, DOI:10.1007/s10853-018-2630-8.
4) A. Zhukov and R. Karlsson, *Journal of Molecular Recognition*, 2007, **20**, 379–385.
5) E. C. Lima, A. A. Gomes and H. N. Tran, *J Mol Liq*, DOI:10.1016/j.molliq.2020.113315.
6) G. W. Peterson, J. J. Mahle, J. B. Decoste, W. O. Gordon and J. A. Rossin, *Angewandte Chemie - International Edition*, DOI:10.1002/anie.201601782.
7) L. Yang, D. Zhang, X. Li, L. Qian, H. Zhang, P. Fang and C. He, *Langmuir*, 2024, **40**, 21395–21406.
8) K. L. Timofeev, S. A. Kulinich and T. S. Kharlamova, *Molecules*, DOI:10.3390/molecules28093916.
